# A Golgi-associated redox switch regulates catalytic activation and cooperative functioning of ST6Gal-I with B4GalT-I

**DOI:** 10.1016/j.redox.2019.101182

**Published:** 2019-04-04

**Authors:** Antti Hassinen, Fawzi Khoder-Agha, Elham Khosrowabadi, Daniela Mennerich, Deborah Harrus, Maxence Noel, Elitsa Y. Dimova, Tuomo Glumoff, Anne Harduin-Lepers, Thomas Kietzmann, Sakari Kellokumpu

**Affiliations:** a University of Oulu, Faculty of Biochemistry and Molecular Medicine, Oulu, Finland; b Université de Lille, CNRS, UMR 8576, UGSF - Unité de Glycobiologie Structurale et Fonctionnelle, F-59000 Lille, France

**Keywords:** Hypoxia, Glycosylation, Golgi homeostasis, Redox state, Sialyltransferase

## Abstract

Glycosylation, a common modification of cellular proteins and lipids, is often altered in diseases and pathophysiological states such as hypoxia, yet the underlying molecular causes remain poorly understood. By utilizing lectin microarray glycan profiling, Golgi pH and redox screens, we show here that hypoxia inhibits terminal sialylation of N- and O-linked glycans in a HIF- independent manner by lowering Golgi oxidative potential. This redox state change was accompanied by loss of two surface-exposed disulfide bonds in the catalytic domain of the α-2,6-sialyltransferase (ST6Gal-I) and its ability to functionally interact with B4GalT-I, an enzyme adding the preceding galactose to complex N-glycans. Mutagenesis of selected cysteine residues in ST6Gal-I mimicked these effects, and also rendered the enzyme inactive. Cells expressing the inactive mutant, but not those expressing the wild type ST6Gal-I, were able to proliferate and migrate normally, supporting the view that inactivation of the ST6Gal-I help cells to adapt to hypoxic environment. Structure comparisons revealed similar disulfide bonds also in ST3Gal-I, suggesting that this O-glycan and glycolipid modifying sialyltransferase is also sensitive to hypoxia and thereby contribute to attenuated sialylation of O-linked glycans in hypoxic cells. Collectively, these findings unveil a previously unknown redox switch in the Golgi apparatus that is responsible for the catalytic activation and cooperative functioning of ST6Gal-I with B4GalT-I.

## Graphical abstract

**Figure:**
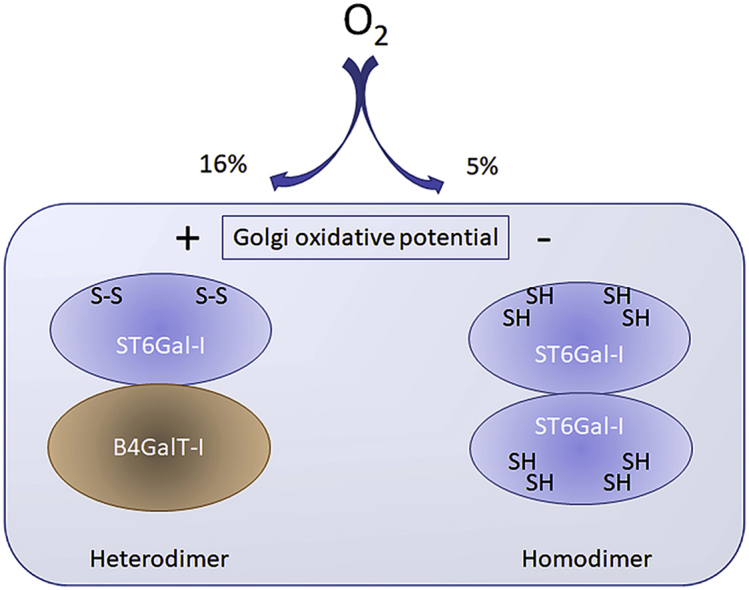

## 1 Introduction

Glycosylation is a ubiquitous modification of cell surface proteins and lipids, and important for a multitude of recognition events between cells, cells and extracellular matrix components, signaling molecules and invading pathogens [1]. Yet, they are dynamic and altered in certain environmental or pathophysiological conditions including cancers, inflammation, and diabetes [[2], [3], [4], [5], [6]], giving rise give to different cellular phenotypes or cell behaviors. This is particularly evident in cancers, in which the more branched and over-sialylated N-glycans as well as truncated O-glycans are common and implicated as being important for tumorigenesis [3,[7], [8], [9], [10]], as they typically increase cancer cell invasion and metastatic spread into adjacent tissues.

Many diseases including those above as well as stroke, myocardial infarction and Alzheimer's disease also show typical changes in cellular redox balance and hypoxic status [[11], [12], [13], [14]]. Hypoxia in turn modulates the functioning of multiple cellular pathways to help cells to survive and proliferate at low oxygen tension. These changes are orchestrated by hypoxia-inducible factors (HIFs), *i.e.* transcription factors that regulate the expression of hundreds of genes affecting among others cellular metabolism and signaling networks [11,15]. Severe hypoxia or HIFs also modulate homeostasis of the endoplasmic reticulum (ER) and the Golgi apparatus (GA). In the former, it typically evokes the unfolded protein response (UPR) [16,17], while in the latter it interferes mainly with Golgi-associated trafficking and glycosylation events [14,[18], [19], [20], [21]]. The observed glycosylation changes often coincide with altered expression levels of certain glycosyltransferase genes, which however, do not always correlate with the glycan profiles displayed by hypoxic cells [22]. Therefore, besides enzyme level changes, other defects must exist and need be identified.

By utilizing lectin microarray-based glycan profiling, we show here that moderate hypoxia (5% O_2_) mainly attenuates terminal sialylation of both N- and O-glycans, given the marked increase in the level of galactose- and N-acetylgalactosamine-terminating glycans (GalNAc-R and Gal-GalNAc-R) in hypoxic cells. Under normal conditions, these glycan epitopes are masked by further sialylation in the Golgi apparatus [8]. Guided by these observations, we chose the B4GalT-I galactosyltransferase and ST6Gal-I sialyltransferase as our target enzymes to define why hypoxia attenuates terminal sialylation of N-glycans. These two enzymes act co-operatively to add terminal galactose and sialic acid to N-glycans by forming a heteromeric complex, a phenomenon that by itself increases enzymatic activity of both complex constituents [23,24]. Our results indicate that of the two enzymes, only the ST6Gal-I is sensitive to hypoxia and is not active in hypoxic cells. Thus, the data unveil a hitherto unknown regulatory circuit that is hypoxia-sensitive, relies on disulfide bond formation, and is needed for catalytic activation of ST6Gal-I in the Golgi apparatus.

## 2 Materials and Methods

### 2.1 Plasmid constructs

All glycosyltransferase expression plasmids were prepared from commercially available cDNA clones (Imagenes GmbH, Berlin, Germany). Golgi-localized pcDNA3-based FRET enzyme constructs possessing C-terminal mCerulean, mVenus or mCherry variants as well as HA epitope-tag were prepared as previously described [24]. The glycosyltransferase genes were inserted in frame with the tags using 5′ *Hind*III, *Bam*HI or *Eco*RI, and the 3′ *Xba*I restriction sites. Golgi localized pH-sensitive and redox-sensitive probes (pHluorin and RoGFP2, respectively) were constructed by adding a cDNA fragment that encodes 80 N-terminal amino acids of the B4GalT-I to their N-termini. The RoGFP2 plasmid constructs were prepared by using the pECFP-ER- and pECFP-Golgi-targeting vectors as templates (Clontech Laboratories Inc.) and by replacing the fluorophore with the RoGFP2 sequence. All constructs were sequence-verified with the ABI3500xL Genetic Analyzer.

### 2.2 RNA preparation and quantitative real-time PCR

Isolation of total RNA was performed using the Qiagen RNeasy^®^ Mini Kit (Qiagen, Switzerland) following the manufacturer's instructions. One μg of total RNA was used for cDNA synthesis with the iScript™ cDNA synthesis Kit (Bio-Rad, Finland). Quantitative real-time PCR was performed using a Real-Time PCR System (*Applied Biosystems 750,* Life Technologies, Finland) and Power SYBR^®^ green PCR master mix (Applied Biosystem Life Technologies, Finland). All primer sets (Expanded view Table S1) were validated for product identity and amplification efficiency using standard dilution and melting curve analyses. β-actin, 18s rRNA and β-d-glucuronidase (GusB) were used as internal controls to normalize the variability in expression levels. The experiments for each data point were carried out in triplicate. The relative quantification of gene expression was determined using the ΔΔCt method [25].

### 2.3 Cell cultivation and treatments

COS-7 cells and the RCC4-pVHL-defective renal cell carcinoma cells and wild type RCC4-pVHL+ cells (with reintroduced pVHL protein) were cultivated in high glucose DMEM/10% FCS as described elsewhere [26]. Cell transfections were done 20 h after plating the cells by using 0.5 μg of each plasmid cDNA and the FuGENE 6™ transfection reagent according to the supplier's instructions (Promega, Fitchburg, WI, USA). 10 h post-transfection, cells were kept either in normoxia (16% O_2_/79% N_2_/5% CO_2_) or transferred to moderate hypoxia (5% O_2_/90% N_2_/5% CO_2_) for 4–48 h before further analyses. When appropriate, cells were also treated at the same time or alone with 40 μM chloroquine or 10–50 mM dithiothreitol (Sigma Aldrich, St. Louis, MO, USA) for 10 min before the measurements.

### 2.4 Cell staining and co-localization studies with fluorescence microscopy

Cells were prepared for immunofluorescence microscopy as follows. After fixation with 2% p-formaldehyde (30 min), cells were permeabilized with 0.1% saponin in PBS and stained with the anti-GM130 (610822, BD Biosciences, San Jose, CA, USA), monoclonal anti-HA (Sigma Aldrich, St. Louis, MO, USA) and polyclonal anti-B4GalT-I (#HPA010807, Sigma Aldrich, St. Louis, MO, USA) antibodies. After washing, cells were stained with relevant species-specific Alexa Fluor 488- and 594-conjugated anti-mouse and anti-rabbit secondary antibodies (Invitrogen, Carlsbad, CA, USA), mounted and imaged using the Zeiss Observer. Z1 microscope equipped with a LSM 700 confocal unit, Zen2009 software (Carl Zeiss AG, Oberkochen, Germany), a 63X Plan-Apo oil-immersion objective and appropriate filter sets for each dye. Cells over-expressing various mVenus or mCherry-tagged enzyme construct were imaged without co-staining.

Co-localization studies were performed in the cells expressing the selected mVenus enzyme constructs. 20 h post-transfection, cells were fixed, and stained with the Golgi marker (GM130) antibody before imaging with the Zeiss LSM700 confocal microscope. In each case, 10 distinct Z-stack image sets from 10 different cells were taken, of which 3 medial sections were used for calculating the Pearson's correlation coefficients (r) using the Zen 2009 software. The r-values are expressed as the mean (±S.D) of 30 different confocal planes for each enzyme construct. T-antigen levels were determined using the Alexa-594 conjugated peanut agglutinin lectin (PNA) as described earlier elsewhere [26]. Briefly, cells were grown on 96- well plates either in normoxia or hypoxia as indicated, and fixed with 2% p-formaldehyde for 30 min. Fixed cells were then quenched with 1% BSA-PBS (pH 7.4) for 1 h in RT. After washing, the cells were stained with Alexa Fluor 594-conjugated peanut agglutinin (3 μg/ml, Invitrogen Inc. Waltham, USA) in the same buffer. Appropriate filter sets for each fluorophore were used.

### 2.5 Lectin microarray analyses

Cells cultivated both under normoxia (16% O_2_) and hypoxia (5% O_2_) for 20 h were lysed on plates (30 m in +4 °C) with the lysis buffer as described previously [27]. After clearing (12,000×*g* at 4 °C for 15 min), 6 μg of total protein in the cleared lysate was labeled with 25 μg of NHS activated DyLight 650 protein dye (Thermo Scientific, Waltham, USA) in 50 μL of labeling buffer for 30 min at 20 °C with constant agitation. The reaction was quenched by adding 50 mM ethanolamine in Tris-HCl/150 mM NaCl buffer for an additional 30 min at RT and cleared by centrifugation (12,000×*g* for 10 min in RT). Labeled samples were then applied onto pre-printed and pre-quenched (50 mM ethanolamine) microarray slides (Nexterion, Schott, Germany), and further incubated in a humidified chamber with constant agitation for 2 h in RT. Slides were then washed 5 times for 5 min each with the assay buffer. After drying, each slide was imaged with Operetta high content imaging system (PerkinElmer Inc., Waltham, USA) using an appropriate filter set for the DyLight 650™ dye. The intensities of bound label were quantified in triplicate from 4 parallel arrays using the ImageJ plug-in macro developed for protein microarray analyses. Two separate sample preparations were used for the glycan profiling. The lectins (45 different lectins, see Suppl. Fig. S1 for their specificities) were purchased from EY Laboratories (San Mateo, CA, USA) or from the Vector Laboratories (Youngstown, OH, USA).

### 2.6 FRET, ratiometric Golgi pH and redox measurements

FRET measurements were performed using the mVenus (mVen) and mCherry (mChe) as a FRET pair. Cell transfections were done according to manufacturer's instructions using the FuGene6™ transfection reagent. In brief, 20 h after the transfections, cells were plated on 96-well culture dishes, allowed to attach for 6 h, and fixed with 4% paraformaldehyde (PFA) before washing with PBS (pH 7,4) and imaging in PBS using the Operetta High Content Imaging System (PerkinElmer) with appropriate filter sets for each fluorescent protein. The Harmony 4.1 software was used for the quantification of the FRET signal (for detailed description of the FRET measurement protocol, see Ref. [28]). For Golgi pH measurements, COS-7 cells expressing the ratiometric pH-sensitive pHluorin were used. Cells stably expressing the Golgi-localized GT-pHluorin were seeded on 96 well microscopy plates in phenol red-free media (FluoroBrite™ DMEM, Thermo Fisher Scientific), and then grown in normoxia and hypoxia for 24 h. The plates were sealed tightly with a plastic cover before imaging with the Operetta High Content Imaging System (PerkinElmer Inc.). Appropriate filter sets for two different excitation wavelengths (420 nm and 470 nm excitation, 500–550 emission) were used to facilitate ratiometric quantification of the signal intensities with the in-build Harmony software package.

Fluorescence intensity ratios were converted to pH values by using an *in situ* calibration curve obtained by using specified pH calibration buffers prepared in 125 mM KCl/20 mM NaCl/1.0 mM CaCl_2_/1.0 mM MgCl_2_. pH of the buffers was adjusted to 7.5, 6.5, and 5.5 using 20 mM HEPES, MOPS, or MES, respectively. All calibration buffers also contained 5 mM nigericin and 5 mM monensin to dissipate monovalent cation gradients. Redox potential measurements were performed similarly, but by using the ratiometric ER- and Golgi-localized RoGFP2 redox probes [29]. Cells were seeded in 96 well plates, transfected with Golgi-RoGFP2 using the Lipofectamine 3000 transfection reagent according to manufacturer's instructions. Cells were grown under normoxia or hypoxia for 24 h. RoGFP2 ratios were quantified in triplicate from the imaged cells as above using the same filter sets as for GT-pHluorin (420 nm and 470 nm excitation, 500–550 emission) and the Harmony software package (PerkinElmer Inc.).

### 2.7 SDS-PAGE and immunoblotting

Western blot analyses were carried out as described elsewhere [26]. In brief, cells were lysed on plates with the lysis buffer (50 mM Tris-HCl, 150 mM NaCl; 1% TX-100; 2 mM EDTA; 2 mM EGTA; 10 mM Na_2_PO_7_, pH 6.5 or pH 7.5, see below) which also contained protease inhibitors (Complete Mini, Roche, Basel, Switzerland). After clearing (12000×*g* for 5 min), 50–100 μg of total protein in SDS-sample buffer was run using 7.5% sodium dodecyl sulfate (SDS)-polyacrylamide gel electrophoresis before immunoblotting with the appropriate antibodies. These included a rabbit polyclonal anti-B4GalT-I (#HPA010807, Sigma Aldrich, St. Louis, MO, USA), mouse monoclonal anti-HIF-1α or HIF-2α antibodies (Abcam, Cambridge, UK), mouse monoclonal anti α-tubulin antibody (Sigma Aldrich, St. Louis, MO, USA), rabbit polyclonal anti-ST6Gal-I antibody (anti-CD75, #AP21150PU-N, Acris antibodies, Herford, Germany), and anti-GlcNAcT-1 antibody (a kind gift from Prof. Pamela Stanley, NY, USA). As secondary antibodies, either anti-mouse or anti-rabbit Fab2-fragments conjugated to horseradish peroxidase (1:10000, Abliance, Compiègne, France) were used. Stained protein bands were visualized using the ECL reagent and the GelDoc instrument (Bio-Rad, Hercules, USA). Quantification of protein bands from the digitalized pictures was done using the ImageLab-software from Bio-Rad.

### 2.8 BODIPY maleimide staining of free thiol groups

COS-7 cells expressing B4GalT-I HA or ST6Gal-I HA were grown in either 20% or 5% oxygen as described above, and then further incubated with 500 μg/ml BODIPY FL maleimide (10 mg/ml in DMF, (Thermo Scientific, Waltham, USA) for 1 h at 20 °C with continuous agitation. The labeling reaction was stopped with the addition of 1.5 M hydroxylamine, pH 8.5, before cell lysis in tetraborate lysis buffer (100 mM, pH 8.5, supplemented with 150 mM NaCl, 1% TX-100, 2 mM EDTA, 2 mM EGTA, 10 mM Na_2_PO_7_). After centrifugation (12,000 × *g* at 4 °C for 15 min), 2 μg of monoclonal anti-HA antibody (Sigma Aldrich, St. Louis, MO, USA) was added to the supernatant and incubated for 1 h before the addition of protein G-Sepharose beads (30 μl/reaction, GE Healthcare, Little Chalfont, UK). After rotating the sample for 24 h, the beads were collected by brief centrifugation, washed 5 times with 50 mM tetraborate buffer, pH 8.5, and eluted from the beads (10 min at 50 °C) using the same buffer. Unbound proteins in the supernatant were precipitated with 10% TCA, collected by centrifugation and washed 5 times with acetone. The eluted and TCA-precipitated samples were suspended in 2× sample loading buffer with 0,2% SDS (pH 6.5) and separated by using a 7.5% blue native PAGE gel using a protocol described by Wittig et al. [30]. The gel was stained with PageBlue dye (Thermo Scientific, Waltham, USA) before imaging with the GelDoc imager equipped with a GFP filter. Band quantification was done using the ImageLab software (Bio-Rad).

### 2.9 Cysteine mutagenesis

ST6Gal-I cysteine mutants C24S, C184A, C353A, C406A and the double mutant (C353A-C406A) were generated by site-directed mutagenesis fusing the ST6Gal-I_WT_ plasmid cDNA and the QuickChange Lightning site-directed mutagenesis kit (Agilent Technologies Inc., Santa Clara Ca, USA). Primers containing one, two or three base pair mutations were used for PCR amplification. XL10 gold *E. coli* strain was used to clone the mutants. The mutated inserts were ligated into the pcDNA3 plasmid containing either C-terminal mVenus or mCherry fluorescent tags using the 5′ cloning site (*Hin*dIII) and the 3′ cloning site (*Xba*I). The clones were selected by using ampicillin resistance as a marker. All plasmids cDNAs were sequence verified before use. Correct Golgi localization of the mutants was also verified in the transfected cells by using confocal microscopy as described above. The used primer sequences for the mutagenesis of selected cysteines in ST6Gal-I are available upon request.

### 2.10 SNA staining of transfected cells

Cell surface α-2,6-linked sialic acid content was determined by using the FITC-conjugated Sambucus Nigra Agglutinin lectin (SNA). COS-7 cells were grown in DMEM supplemented with 10% FBS (HyClone, Thermo Scientific, Waltham, MA, USA). One day after plating, cells were detached and transfected using electroporation with 2.5 μg of each plasmid cDNA according to the supplier's protocol (Nucleofector™, Lonza, Basel, Switzerland). 24 h post-transfection, cells were transferred to a 96-well Ultra™ plate (PerkinElmer Inc., USA), allowed to attach, and cultivated for 24 h at both normoxic and hypoxic conditions in the presence of neuraminic acid (10 mM, Sigma-Aldrich). Cells were then fixed with 2% p-Formaldehyde, quenched with 1% BSA-PBS before staining with FITC-conjugated SNA-lectin (1 μg/ml, Vector Laboratories Ltd, UK) for 1 h. Before final washing with PBS and imaging using the high content imaging system (Operetta, PerkinElmer Inc.). Cell nuclei were counter-stained with the Hoechst dye to allow selection of individual cells for quantification. For normalization, identical cell samples were subjected to SDS-PAGE and western blotting with the anti-CD75 antibody (#AP21150PU-N, Acris Antibodies GmbH, Germany) to determine the level of ST6Gal-I protein in each sample (Suppl. Fig. S5).

### 2.11 Sialyltransferase activity assays

COS-7 cells were transfected with the selected plasmids using electroporation as described above. Each transfected COS-7 cell pellet (100,000 cells) were suspended in 20 μL PBS containing protease inhibitor (complete Tablets *EASYpack*, Roche), and 20 μL RIPA buffer (10 mM Tris HCl; 150 mM NaCl; 1% Triton X-100; pH 6.4). Sialyltransferase activity measurements were carried out using desialylated fetuin as an acceptor essentially as described earlier [31]. In brief, the reaction mixture (30 μL, containing 40 mM cacodylate buffer (pH 6.2), 4 mM MnCl_2_, 0.08%, Triton CF-54 (Sigma-Aldrich), 50 μM CMP-Neu5Ac (13.6 μM CMP-[^14^C]Neu5Ac (22.5 nCi, 50,000 dpm) and 36.07 μM cold CMP-Neu5Ac), 20 mg/ml desialylated fetuin and enzyme sample (6 μL)) was incubated at 37 °C for 16 h. The reaction was stopped by precipitation of glycoproteins with phosphotungstic acid (PTA; 1 ml, 5% in 2 N HCl) followed by filtration on Whatman GF/A glass microfiber filters. Incorporated radiolabeled sialic acid into glycoproteins was quantified by liquid scintillation counting in UltimaGold (3 ml; PerkinElmer) using a Hidex 300 SL counter. Identically transfected cell samples were used for SDS-PAGE and western blotting with the ST6Gal-I specific antibody to allow normalization.

### 2.12 Cell proliferation, wound healing and apoptosis assays

COS-7 cells were transfected with the ST6Gal-I_WT_ and the ST6Gal-I_CysDM_ constructs using electroporation (Lonza) according to manufacturer's instructions. An empty vector (pCDNA 3.1) was used as a control. Cells were then plated into 96 well cell culture plates (5 × 10^3^ cells/well for cell proliferation assay and SNA staining; 5 × 10^4^ cells/well for scratch wound healing assay and 1 × 10^4^ cells/well for apoptosis assays; 8 wells/each assay). 8 h later, neuraminic acid (10 mM) was added to cells before further analyses. The following day, cells were stained with the SNA lectin to confirm sialic acid content of cell surface glycans. Cell proliferation rate was followed using the IncuCyte^®^ ZOOM System (Essen BioScience) with live phase contrast recording of cell confluence for 5 days in 3 h intervals. Confluent cell monolayers growing on Essen ImageLock Plates (Essen Bioscience) with 1% serum, were also subjected to “scratch wound healing” assay by making a wound with the 96 PTFE pin Wound Maker (Essen Bioscience). Wound closure was followed thereafter using phase contrast-imaging for 120 h in 3 h intervals. In both assays, the confluence analysis was performed using the basic IncuCyte software settings. Apoptosis assay was performed by treating cells on Cell Carrier Ultra plates with staurosporine (5 μM) and by following nuclear fragmentation and morphology with the Operetta High Content Imaging system (PerkinElmer Inc.) during the next 6 h of incubation. Nuclear fragmentation, morphology and area were measured using the ready-made protocols present in the Harmony™ software package.

### 2.13 Statistical analyses

Statistical analyses were performed using two-tailed Student's t-test for the comparison of small, normally distributed samples. Only statistically significant changes are marked in the figures with stars as follows: p < 0.05*, p < 0.01**, p < 0.001***.

## 3 Results

### 3.1 Hypoxia inhibits terminal sialylation of both N- and O-linked glycans

**Fig. 1:**
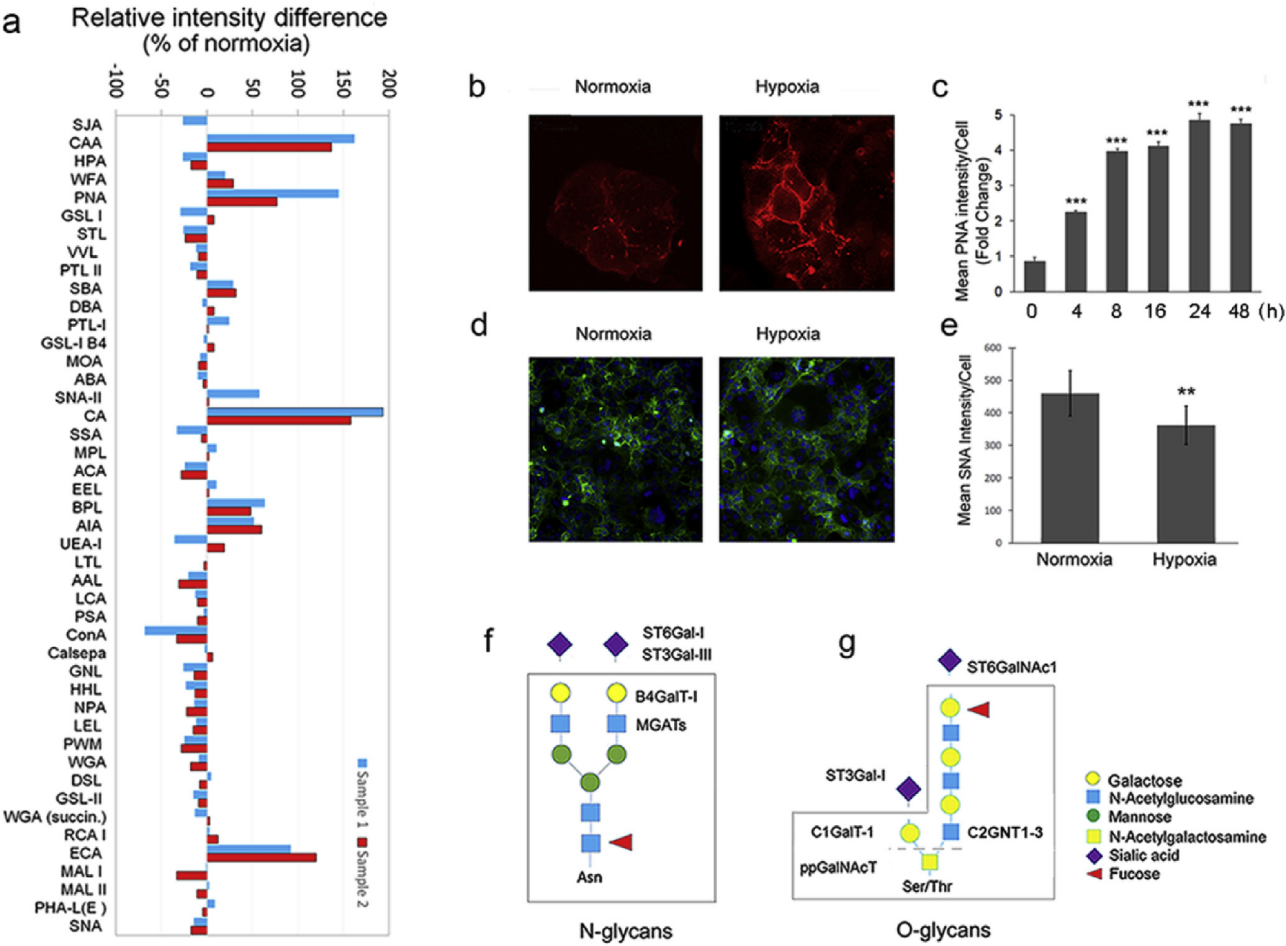
Hypoxia-induced glycosylation changes in COS-7 cells. a) Lectin microarray glycosylation profiling in normoxic and hypoxic cells. The graph shows the relative intensity differences (subtracted fingerprints) between two distinct hypoxic and normoxic samples (blue and red bars) for each lectin (X-axis labels, for raw data and sugar binding specificities, see Expanded View Fig. 1). The differences in binding of sample glycans to each lectin were calculated from the background corrected raw data values and are expressed as percentages from normoxic values. Positive values depict up-regulation and negative values down-regulation. **b)** Fluorescence microscopy images of the cells stained with the Alexa 594-conjugated PNA lectin. **c)** Quantification of the bound PNA lectin to cells at different time points during hypoxia exposure. Quantification was done using a high content imaging system (Operetta, PerkinElmer Inc.). Each bar represent the mean PNA intensity/cell (±SD, n = 3, 90,000 cells each) as fold changes from normoxic cells. **d**) Representative figures of cells stained with Alexa 488-conjugated SNA lectin to quantify the α-2,6-linked sialic acid levels in normoxic and hypoxic cells. **e**) Quantification of the bound SNA lectin using the high content imaging system in triplicate (mean/cell ±SD, 90,000 cells each). In all figures, only statistically significant changes are marked by stars (p < 0.05*; p < 0.01**; p < 0.001***), Insignificant changes (p > 0.05) are unmarked. **f-g)** Schematic representation of the N- (f) and O-linked (g) glycosylation changes detected in hypoxic cells (boxed areas) by lectin microarray analyses. The main enzyme names affected by hypoxia are depicted outside the boxed areas.

To investigate first how hypoxia alters cellular N- and O-linked glycosylation profiles, we used a lectin microarray-platform with 45 different lectins. We found that hypoxia exposure of cells induced marked changes in both N- and O-linked glycans (Fig. 1a), increasing mainly the level of terminal galactose residues (detected with the ECA lectin). We also detected a small but consistent decrease in high-mannose type N-glycans (detected with ConA, GNL, HHL, LEL and PWM lectins). Moreover, the level of truncated mucin-type O-glycans were increased, especially the Tn- (GalNAc-ser/thr) and the T- (Galβ1,3GalNac-) antigens (detected with the CA, CAA, PNA, BPL and AIA lectins). The increase in the T-antigen level was detectable also in cells stained with Alexa 594-conjugated peanut agglutinin (PNA, Fig. 1b). This increase was detected already after 4 h of hypoxia exposure (Fig. 1c). Maximal (5-fold) increase was seen with 24 h hypoxia treatment without detectable decrease by 48 h time point. Consistent with these findings, we saw a small but significant reduction (30%) in α-2,6-linked sialic acid levels in hypoxic cells at 24 h time point by using both lectin microarray glycan profiling and by staining of the cells with the Alexa 488-conjugated SNA lectin (Fig. 1d and e). Collectively, these data indicate that hypoxia attenuates mainly terminal sialylation of both N- and O-glycans via affecting ST6Gal-I, ST3Gal-III sialyltransferases that act on N-glycans (Fig. 1f), or ST3Gal-I (SIAT4A) and ST6GalNAc1 (SIAT7A) sialyltransferases that both act on O-glycans (Fig. 1g). The former is also implicated in the synthesis of keratan sulfate and glycolipids (see the Kegg database at: https://www.genome.jp/kegg/).

### 3.2 Hypoxia induces sialylation defects in a HIF- and Golgi pH-independent manner by lowering Golgi oxidative potential

**Fig. 2:**
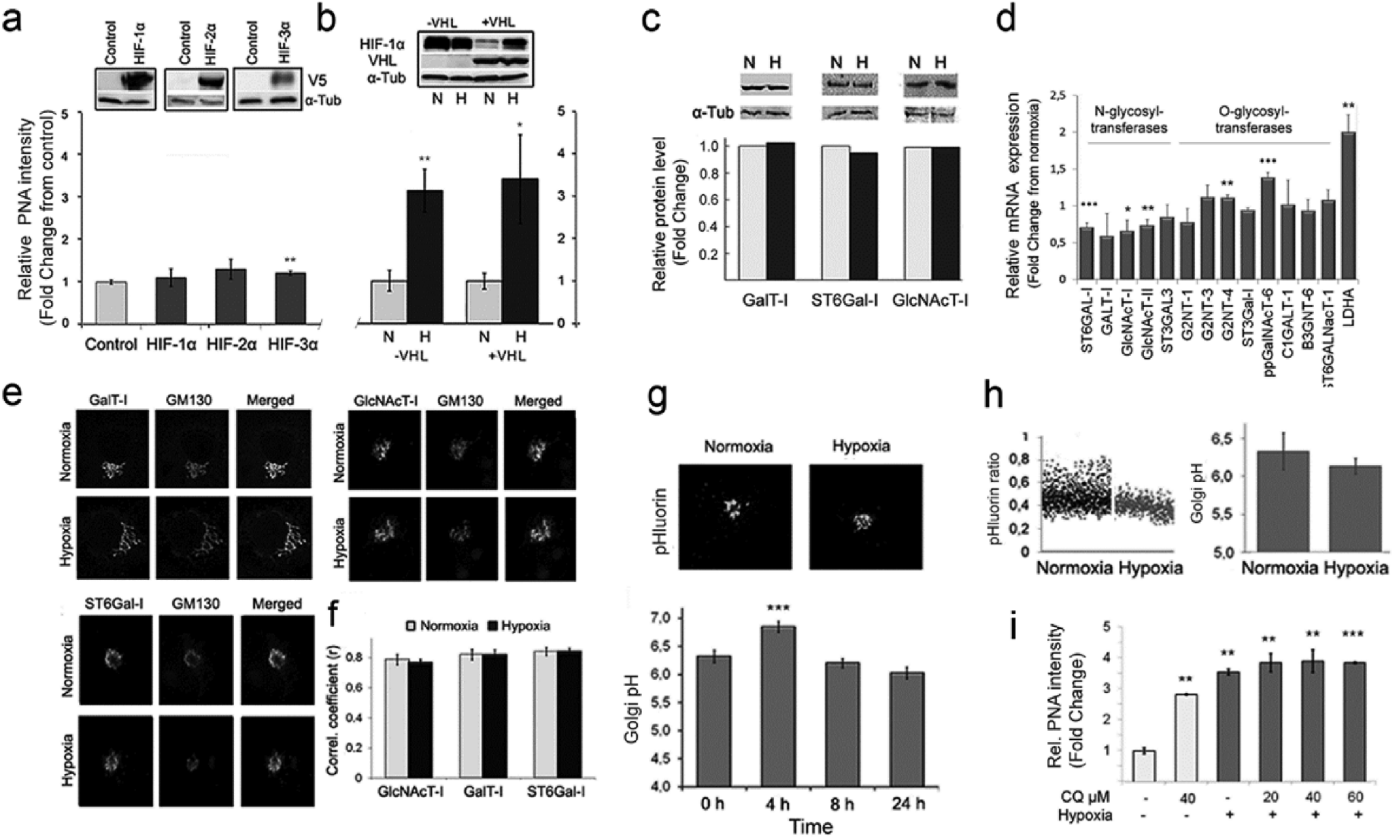
The effect of Hypoxia/HIFs on PNA staining and Golgi redox state changes in normoxic (N) and hypoxic (H) cells. **a)** PNA binding to normoxic COS-7 cells overexpressing HIFs (-1α, -2α, -3α). Cells expressing the depicted V5-taged HIF isoforms were stained with Alexa 594-conjugated PNA lectin before quantification of the bound lectin in triplicate (mean/cell ± SD, 90,000 cells/assay) with the high content imaging system. Insets show representative immunoblots for the HIF isoforms probed with V5-tag antibody. α-tubulin served as a loading control. **b)** PNA binding to VHL-deficient RCC4 and RCC4-reconstituted (RCC4^pVHL^) cells. Cells grown under normoxia and hypoxia were stained as above, and the PNA binding intensities were measured in triplicate as above. The changes are expressed as fold changes from normoxic control cells using the same scaling for comparison**).** The inset shows a representative Western blot probed with VHL protein-specific antibodies. (N, normoxia; H, hypoxia). Statistically significant changes are marked with stars (p < 0.05*, p < 0.01**, p < 0.001***). **c-e**) The effect of hypoxia on enzyme protein and mRNA levels, and their subcellular distribution as well as on Golgi pH homeostasis. **c**) Immunoblotting analyses of enzyme protein levels in COS-7 cells grown either in normoxia (N) or hypoxia (H). The samples were subjected to immunoprecipitation and then immunoblotting with the HA-tag specific antibodies. The band intensities were quantified to allow estimation of their relative amounts in normoxic and hypoxic cells. α-tubulin was used as a loading control. **d**) Quantitative RT- PCR analyses on the glycosyltransferases mRNA expression levels in the cells. mRNA levels were determined as described in “Materials and Methods” section. The values are expressed as fold changes from normoxic samples. Statistically significant changes are marked by stars (p < 0.05*, p < 0.01**, p < 0.001***). **e**) Subcellular distribution of the depicted enzymes in normoxic and hypoxic cells. Cells were transfected with the indicated mVenus-enzyme constructs, cultivated and processed for confocal immunofluorescence microscopy 24 h post-transfection and Z-stack imaging. Representative figures are shown for each enzyme together with the Golgi marker protein GM130 (red, stained with the protein specific antibody and Alexa 594-conjugated secondary antibody). **f**) Z-stack images were taken from 10 different Golgi elements (30 images per one Golgi area) to allow calculation of the Pearson's correlation coefficients. The Zen software (Zeiss, Jena, Germany) with an add-in module was used for the calculations. Statistically significant changes over the control are marked in the figures with stars (p < 0.05*, p < 0.01**, p < 0.001***). **g-i**) Golgi luminal pH in normoxic and hypoxic cells. Golgi pH was determined in cells expressing the Golgi-localized ratiometric pHluorin with the high content imaging system. The ratios were transformed to pH values (mean ± SD, n = 3, 15,000 cells each) using the formula derived from a pre-determined calibration curve (for details, see the “Materials and Methods” section in the Supplementary Appendix). **g**) Localization of the pH-sensitive probe (pHluorin) in the Golgi apparatus and time-dependent changes in the mean Golgi resting pH in hypoxic cells. **h)** Golgi resting pH determined in single cells in cells grown either in normoxia and hypoxia. Each dot in the graph (left) denotes to a single cell, while the bars (right) represent their mean values (±SD, n = 3, 15,000 cells each). The measurements were carried out 24 h post-transfection. **i**) Combined effects of chloroquine and hypoxia on PNA lectin binding in normoxic and hypoxic cells. Cells treated with or without the compound (Chloroquine, CQ, 40–60 μM) were grown in normoxic (light bars) and hypoxic (dark bars) conditions for 24 h before fixation, staining with the Alexa-594 conjugated PNA lectin and quantification of the bound lectin with the Operetta high content imaging system. The bars denote to fold changes (mean PNA intensity ± SD, n = 3, 15,000 cells each) from control cells (no CQ, normoxia). Statistically significant changes over the control are marked in the figures with stars (p < 0.05*, p < 0.01**, p < 0.001***).

Since the response to hypoxia is normally orchestrated by hypoxia-inducible factors (HIFs) [11], we tested next whether the above sialylation defects are HIF-dependent by individually over-expressing each of the three known HIF isoforms (HIF-1α, -2α, and -3α). Measurement of the T-antigen levels in the cells with the PNA lectin showed that none of them increased the T-antigen levels in the cells (Fig. 2a). To verify this, we switched the experimental set up and utilized the von Hippel-Lindau protein (pVHL)-deficient renal carcinoma cells (RCC4^−pVHL^), where HIF-1α levels are always upregulated regardless of whether the cells are grown under normoxia or hypoxia (Fig. 2b) [32,33] For comparative reasons, we also used RCC4 cells with reintroduced pVHL protein (RCC4^pVHL^). In these cells, HIF levels are up-regulated only by hypoxia. Both RCC4 cells and the reconstituted RCC4^pVHL^ cells displayed low levels of the T-antigen in normoxia (Fig. 2b). In contrast, they both showed marked (3-fold) increase in PNA binding in response to 24 h hypoxia. These data indicated that the observed sialylation changes are not mediated by the accumulation of HIFs, but rather, involve a HIF-independent mechanism(s).

Therefore, we next focused on other potential defects by which hypoxia could attenuate terminal sialylation of N- and O-glycans. As our main targets, we chose N-glycan terminating ST6Gal-I sialyltransferase and its binding partner β1,4-galactosyltransferase (B4GalT-I or GalT-I) due to their co-operational functioning [23]. Immunoprecipitation and immunoblot analyses (Fig. 2c) showed that 24 h hypoxia exposure did not induce marked expression level changes of these two endogenously expressed enzyme protein, nor that of GlcNAcT-I (a preceding enzyme in N-glycan synthesis), despite the fact that qRT-PCR analyses revealed a slight down-regulation of their mRNAs as well as an up-regulation of O-glycan modifying ppGalNAcT-6 mRNA together with the HIF-target gene LDHA mRNA (Fig. 2d). This indicated that potential expression changes in the ST6Gal-I cannot be responsible for the loss of sialylation in hypoxic cells. Z-stack imaging with a confocal fluorescence microscopy revealed that hypoxia also did not alter the subcellular distribution of these three enzymes (Fig. 2e). In each case, the Pearson's correlation coefficients were remarkably similar between normoxic and hypoxic cells, relative to the GM130 cis-Golgi marker (Fig. 2f). Moreover, since glycosylation is a pH-sensitive process [34] and hypoxia is known to induce a metabolic shift to glycolysis and thereby increase acid load [35,36], we also tested whether hypoxia can cause sialylation defects by altering Golgi luminal pH (Fig. 2g–i). Golgi pH measurements using a ratiometric pH-sensitive probe pHluorin [37] revealed a transient pH increase at 4 h of hypoxia treatment (Fig. 2g), which, however, rapidly declined (by 8 h) and reached the original level at 24 h (pH 6.3 vs. 6.1, respectively, Fig. 2g). At steady state, Golgi resting pH in hypoxic cells was not significantly lower than that of normoxic cells (Fig. 2h). Finally, combined treatments of cells with hypoxia and 40 mM chloroquine (CQ, a pH gradient dissipating agent, see Ref. [26] were not cumulative, despite they both increased PNA binding by ∼3 fold (Fig. 2i). Taken together, these data indicate that reduced sialylation of N-glycans is not associated with changes in the expression and localization of the enzymes involved, nor with changes in Golgi luminal pH.

**Fig. 3:**
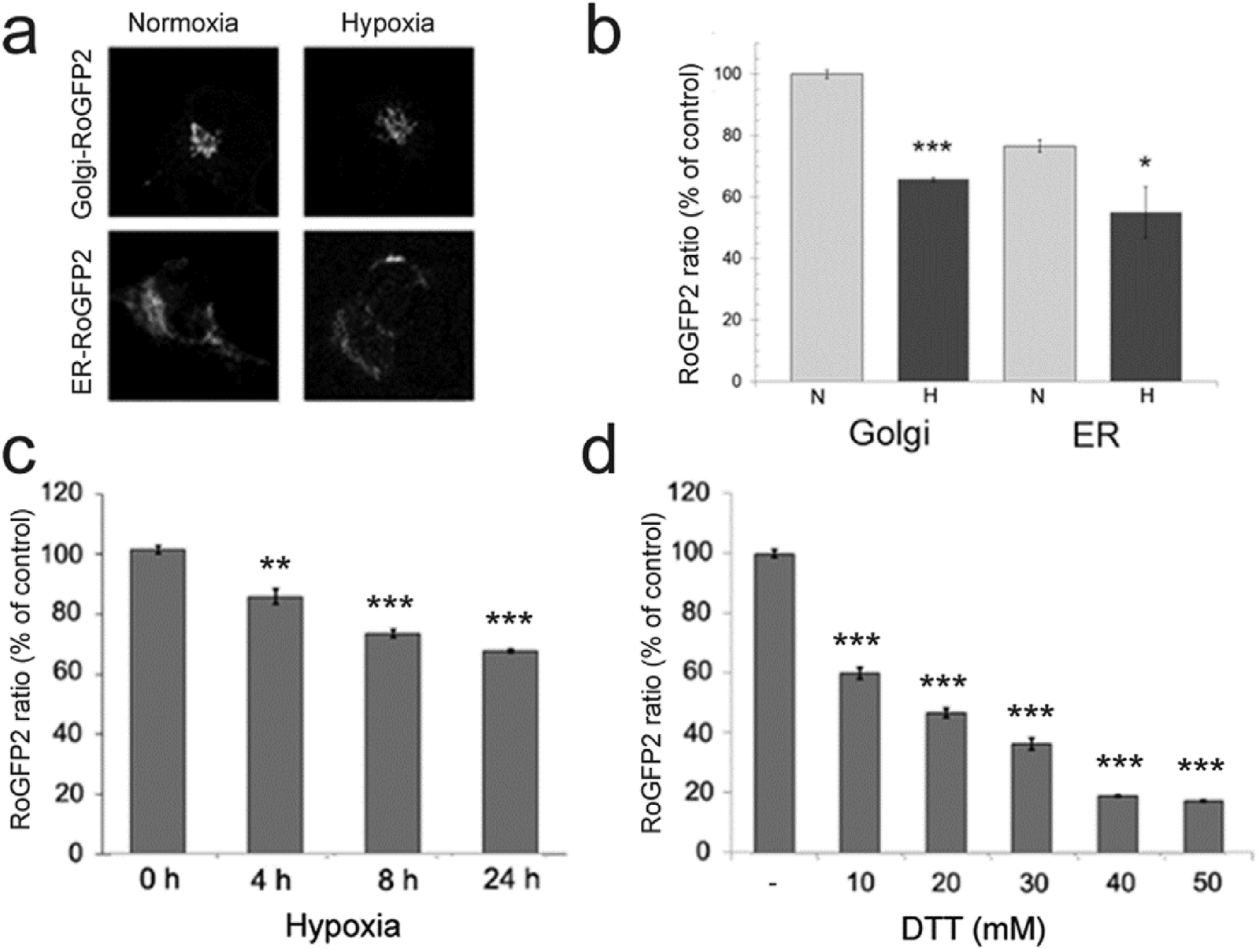
Determination of redox state of the Golgi apparatus and the endoplasmic reticulum (ER). **a**) The figures show cells expressing the Golgi- (top) and ER-localized (bottom) ratiometric RoGFP2 redox sensitive probe both in normoxic and hypoxic cells (for more details, see the “Materials and Methods”-section. **b)** Quantification of the RoGFP2 ratios (a measure of redox state) in the cell using the high content imaging system. The bars denote to the mean RoGFP2 ratios expressed as percentages from normoxic values (±SD, n = 3, 10–15000 cells each). Only statistically significant changes relative to normoxia are marked by stars (p < 0.05*, p < 0.01**, p < 0.001***). N, normoxic cells; H, hypoxic cells. **c)** Hypoxia-induced changes in the Golgi RoGFP2 ratios with time. The ratios were determined as above and are expressed as percentages (mean + SD, n = 3, 10,000 cells each) from the control value (0 timepoint). **d)** Titration of the Golgi redox state with a reducing compound (DTT). Cells were transfected with the Golgi-localizing RoGFP2 plasmid and 20 h later, treated for 10 min with different DTT concentrations (0–50 mM) before determination of the RoGFP2 ratios as above. The mean ratio values (±SD, n = 3, 10,000 cells each) were then used to calculate their percentages from the control (no DTT) values.

Since hypoxia can potentially alter the redox state of a cell, compartment, or protein, we utilized next the ratiometric redox-sensitive fluorescent probe RoGFP2 (Fig. 3a) to examine whether hypoxia alters oxidative potential of the Golgi lumen and also that of the endoplasmic reticulum (ER). We found that the ratio between oxidized and reduced RoGFP2 (a measure of oxidative potential of the environment) in the Golgi in normoxic cells was higher than that of the ER (Fig. 3b), consistent with previous data [38]. We also noted that hypoxia caused a more significant decrease in the RoGFP2 ratio of the Golgi than in the ER (35% vs 25%). This decrease was time-dependent (Fig. 3c), in accord with the increase of the T-antigen expression by hypoxia (Fig. 1c). Next, to estimate the amount of reducing equivalents generated by hypoxia in the Golgi, we titrated Golgi oxidative potential with increasing concentrations of dithiothreitol (DTT). DTT, even at low concentration (5 mM), can confer redox-sensitive disulfide bonds to free thiols [39]. Accordingly, DTT markedly decreased the RoGFP2 ratio in the cells in a concentration dependent manner (Fig. 3d), 8.7 mM DTT giving an equivalent decrease in Golgi oxidative potential to that seen by moderate (5% O_2_) hypoxia.

### 3.3 Hypoxia and DTT inhibit the assembly of heteromeric enzyme complexes in the Golgi

**Fig. 4:**
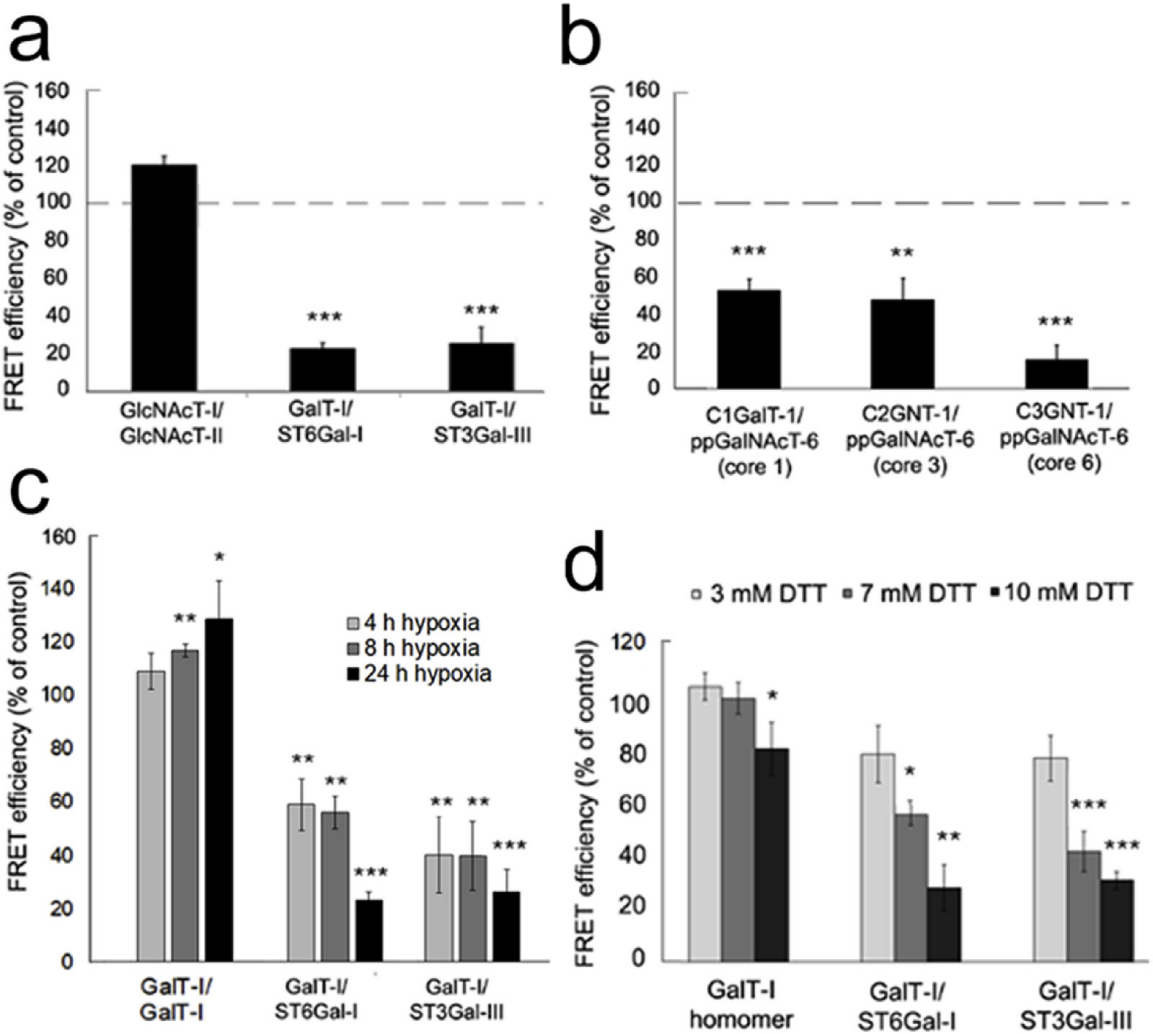
The effect of hypoxia on the assembly of the heteromeric glycosyltransferase complexes in COS-7 cells. **a)** Heteromeric N-glycosyltransferase complexes. **b)** Heteromeric O-glycosyltransferase complexes. In both cases, cells were transfected with the indicated mVenus and mCherry enzyme constructs. The enzyme pairs were chosen based upon their previously established interactions (Hassinen et al., 2011). Thereafter, cells were cultivated in both normoxic and hypoxic conditions for 24 h before quantification of the FRET signals with the high content imaging system. FRET efficiencies were calculated as described in the “Materials and Methods” section, see the Supplementary Appendix) and expressed as percentages (mean ± SD, n = 3, 15,000 cells each)) from the normoxic (control) values (set to 100%). Only statistically significant changes from the normoxic control cells are marked with stars (p < 0.05*, p < 0.01**, p < 0.001***). **c**. Time dependent changes on the assembly of the depicted enzyme complexes by moderate hypoxia. Cells were grown in hypoxic conditions (5% O_2_) and imaged at the indicated times (0, 4, 8, 24 h) before determining the FRET efficiencies for each enzyme pair. The data are expressed as percentages (mean ± SD, n = 3, 15,000 each) from the control (0-time point) values. Statistically significant changes are marked with stars (p < 0.05*, p < 0.01**, p < 0.001***). **d)** Sensitivity of the enzyme complexes to reducing agent (DTT). Cells were processed as above except that they were grown in the presence of different concentrations of DTT for 20 h before quantification of the FRET efficiencies. The data are expressed as percentages (mean % + SD, n = 3 (15,000 each) from the control (no DTT) values. Statistically significant changes are marked with stars (p < 0.05*, p < 0.01**, p < 0.001***).

Previous studies have shown that sequentially acting Golgi glycosyltransferases can form both homomeric and functionally more important heteromeric complexes *in vivo* [23]. Since the formation of heteromers is also sensitive to existing environmental conditions, we decided next to test whether the lowered Golgi oxidative potential could modulate their assembly in the Golgi, and thereby attenuate sialylation. To accomplish this, we utilized the existing mVenus- and mCherry-tagged N- and O-glycosyltransferase constructs as FRET pairs [23]. Quantification of the FRET efficiencies with Operetta high content imaging system revealed that hypoxia had only minor effects on the assembly of enzyme homomers (Fig. EV2a,b). In contrast, hypoxia repressed significantly the assembly of ST6Gal-I/B4GalT-I and ST3Gal-III/B4GalT-I enzyme heteromers (Fig. 4a), enzyme heteromers while having no effect on the assembly of the medial-Golgi GlcNAcT-I/GlcNAcT-II heteromers. Hypoxia also markedly suppressed the assembly of ppGalNAcT-6/C1GalT-I, ppGalNAcT-6/C2GNT-1 (also termed GCNT1) and ppGalNAcT-6/C3GNT-1 (B3GNT6) O-glycosyltransferase heteromers (Fig. 4b). This repression was evident already after 4 h exposure to moderate hypoxia (Fig. 4c). With time, this repression became more prominent and reached 80% inhibition by 24 h of hypoxia treatment (Fig. 4c). Again, no decrease in the formation of homomers (B4GalT-I, also termed GalT-I) was observed. To confirm whether this repression is due to reduced oxidative potential of the Golgi lumen in hypoxic cells, we treated cells with 3–10 mM DTT for up to 24 h in normoxic conditions (Fig. 4d). Similar to hypoxia exposure, this treatment inhibited the assembly of B4GalT-I/ST6Gal-I and B4GalT-I/ST3Gal-III heteromers, without affecting markedly the assembly of B4GalT-I homomers (Fig. 4d) or the subcellular localization of ST6Gal-I (Fig EV3). Taken together, these data show that the hypoxia-induced Golgi redox state change results in the loss of functionally important heteromeric complexes between B4GalT-I and ST6Gal-I and also between B4GalT-I and ST3Gal-III.

### 3.4 Altered Golgi oxidative potential inhibits disulfide bond formation within the catalytic domain of ST6Gal-I

**Fig. 5:**
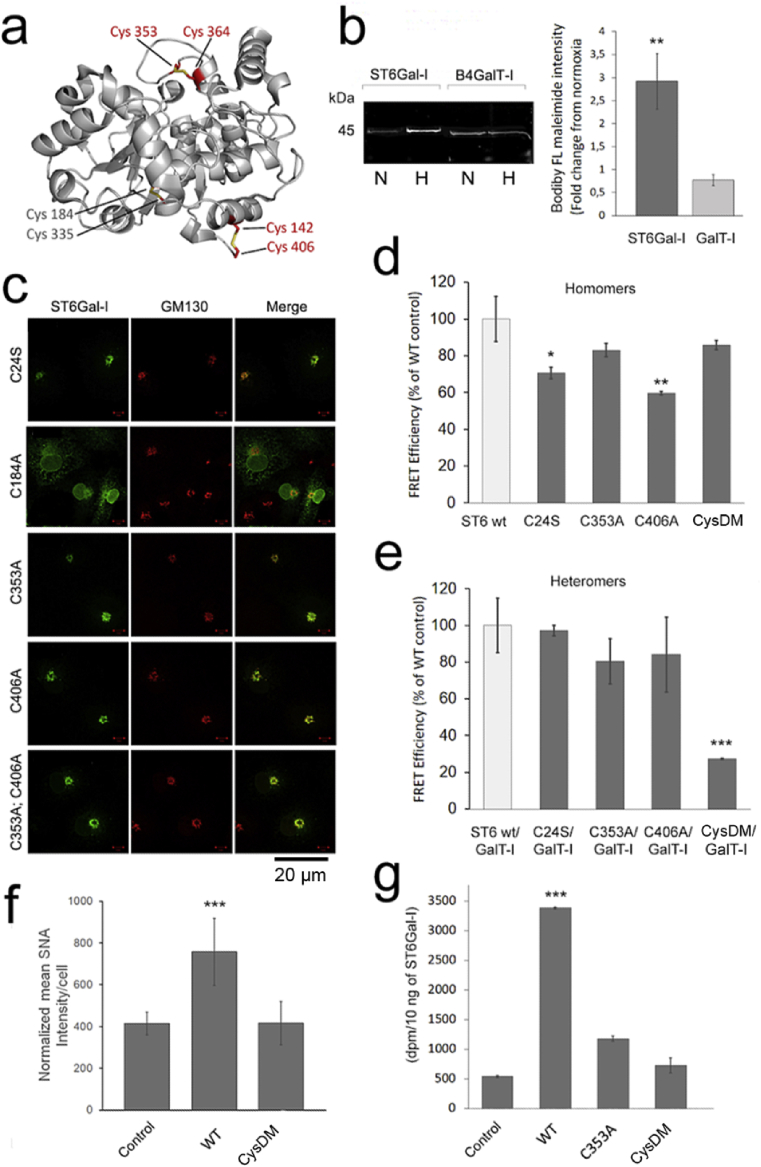
The effect of hypoxia on the formation of surface exposed disulfide bonds in ST6Gal-I catalytic domain. **a)** The crystal structure of the human ST6Gal-I catalytic domain (PDB code 4JS2) showing the 3 disulfide bonds between the depicted cysteine residues. Red text refers to surface exposed disulfide bonds while that grey text depicts an internal disulfide bond. **b)** Bodipy FL-maleimide staining of both ST6Gal-I and B4GalT-I proteins after growing cells for 20 h in normoxic or hypoxic conditions. The figure shows a representative BN-PAGE gel result after labeling of the cells and immunoprecipitation ST6Gal-I and B4GalT-I enzyme proteins with anti-HA tag antibodies from cell lysates prepared from normoxic (N) or hypoxic (H) cells). Band intensities were quantified from digitalized gel images using the ImageJ software. The bars (right) represent fold changes from the normoxic values (set to 1). Statistically significant changes relative to normoxic controls are marked with stars (p < 0.05*, p < 0.01**, p < 0.001***). **c)** Subcellular distribution of the various ST6Gal-I cysteine mutants. Cells were transfected with the indicated mVenus-tagged enzyme constructs, fixed and co-stained with the anti-GM130 antibody (Golgi marker) before imaging with the fluorescence microscopy. All constructs except the C184A colocalized well with the Golgi marker GM130. **d-e)** The effect of cysteine mutations on the assembly of the homomeric (d) and heteromeric (e) ST6Gal-I and B4GalT-I complexes. Cell treatments and quantification of the FRET efficiencies were done as above (for details, see the “Material and Methods” section). The bars denote to mean FRET efficiencies (% ± SD, n = 3, 15,000 cells each) from the wild type control values (set to 100%). Only statistically significant changes are shown and marked with stars (p < 0.05*, p < 0.01**, p < 0.001***). **f)** Quantification of the α-2,6-linked sialic acid levels by SNA lectin staining in control cells and cells expressing the ST6Gal-I_WT_ or the ST6Gal-I_CysDM_ mutant (C353, C406). In brief, 24 h post-transfection cells stained with the Alexa 488-conjugated SNA lectin and with the Hoechst dye to identify cells before quantification of the bound SNA lectin with the Operetta high content imaging system. The data are expressed as the mean SNA intensity/cell (±SD, n = 3, 15,000 cells each) after normalizing the intensities against the ST6Gal-I protein present in the cell lysates (EV Fig. 6). Statistically significant changes relative to control cells (mock-transfected cells) are marked with stars (p < 0.05*, p < 0.01**, p < 0.001***). **g)** Enzymatic activity of the ST6Gal mutants. Cells transfected with the indicated ST6Gal-I variants were lysed in RIPA buffer 24 h post-transfection, after which their activities were determined as described in the “Materials and Methods” section using asialofetuin as an acceptor. The values shown (bars) are expressed as the mean dpm/10 ng ST6Gal-I protein (n = 3) after normalizing them against the ST6Gal-I protein present (determined by immunoblotting as above). Statistically significant changes relative to control cells (mock-transfected cells) are marked with stars (p < 0.05*, p < 0.01**, p < 0.001***).

Next, we investigated the possibility that the lowered Golgi oxidative potential in hypoxic cells may inhibit disulfide bond formation either in the ER or the Golgi compartment using the ST6Gal-I/B4GalT-I pair as a target. The main reasons behind this was their verified catalytic domain interactions [24], the availability of validated antibodies and their published crystal structures [40,41]. Of these two enzymes, ST6Gal-I harbors nine distinct cysteine residues [42], six of which reside in the catalytic domain. Two of these (C184, C335) form one internal disulfide bond that likely is important for the overall structural fold of the catalytic domain. The rest four cysteines form two distinct surface exposed disulfide bonds (Fig. 5a). To test whether any one(s) of these are affected by hypoxia, we stained cells overexpressing both ST6Gal-I and B4GalT-I in both normoxic and hypoxic conditions with BODIPY^®^ FL-conjugated maleimide (a thiol-specific labeling reagent). Quantification of the bound fluorescent dye to either enzyme protein in immunoprecipitated samples after separation with Blue-Native (BN)-PAGE (Fig. 5b) revealed that hypoxia increased labeling of the ST6Gal-I band by 3-fold without affecting labeling of the B4GalT-I. This increase corresponds to the presence of four additional thiol groups in ST6Gal-I protein, given that the enzyme normally has two thiol groups in its transmembrane (TM) domain [42], but together with four additional ones in the catalytic domain (CD), they will give a ratio of 3 (6/2) between normoxic and hypoxic samples. This value is very close to the 2.92 (±0.61) fold increase obtained experimentally in dye binding to STT6Gal-I protein in hypoxic cells (Fig. 5b). Therefore, it is likely that hypoxia causes the loss of two disulfide bonds in the catalytic domain of ST6Gal-I protein.

To reveal which one(s) of the disulfide bonds are affected by hypoxia, we replaced one cysteine in each disulfide bond with serine or alanine residue before testing whether the mutants still localize correctly in the Golgi membranes and can form ST6Gal-I homomers and heteromers with B4GalT-I. We found that only the C184A mutant mislocalized into the ER, likely due to improper folding of the mutant enzyme (Fig. 5c). This is in line with this cysteine forming an internal disulfide bond important for the ST6Gal-I catalytic domain structure (Fig. 5a). All the other single mutants (C24S, C353A, C406A) localized correctly in the Golgi membranes, indicating their proper folding and transport to the organelle. They also did not drastically inhibit the assembly of ST6Gal-I homomers (Fig. 5d), nor the assembly of B4GalT-I/ST6Gal-I heteromers (Fig. 5e). However, the double cysteine mutant (C353A, C406A; termed CysDM) in which the two cysteines forming different disulfide bonds were mutated at the same time showed markedly reduced ability (80% inhibition, Fig. 5e) to interact with B4GalT-I without affecting the assembly of CysDM homomers (Fig. 5d). Thus, the CysDM mutant behaves similarly to the wild type enzyme in hypoxia, suggesting that the two disulfide bonds normally present are critical for the functional assembly of the B4GalT-I/ST6Gal-I heteromers, but not for the assembly of ST6Gal-I homomers. The observed loss of ST6Gal-I heteromers therefore can be responsible for attenuated sialylation of N-linked glycans in hypoxic cells.

To find out whether the loss of the two disulfide bonds (between C353/C364 and C142/C406) has any effect on the enzymatic activity of the ST6Gal, we measured first its ability to add α-2,6-linked sialic acid to cell surface glycans in normoxic cells. The wild type ST6Gal-I (STT6Gal-I_WT_) was used as a control. We expressed both these enzyme constructs individually in the cells for 24 h and determined a-2,6-linked sialic acid content by staining cells with Alexa 488-conjugated SNA lectin and quantifying the bound dye with Operetta high content imaging system. We found that expression of wild type ST6Gal-I increased SNA lectin binding to cells by two-fold, when compared to non-transfected cells (Fig. 5f). However, no such increase was detected with the CysDM, suggesting that this double mutant is enzymatically inactive. We verified this by determining sialyltransferase activity for both the wild type ST6Gal-I and the CysDM. The C353A single cysteine mutant was also included, as this cysteine residue seems to be part of the active site. Cell lysates prepared from cells expressing the ST6Gal-I_WT_ showed roughly 7-fold increase in sialyltransferase activity compared to mock-transfected cells (Fig. 5g). In contrast, the activity of the single mutant (C353A) was only 2-fold higher than that of mock-transfected control cells, while the CysDM showed barely no activity at all. These data demonstrate that the two surface exposed disulfide bonds in ST6Gal-I are not only needed for heteromer assembly (Fig. 5e), but also for its catalytic activity.

**Fig. 6:**
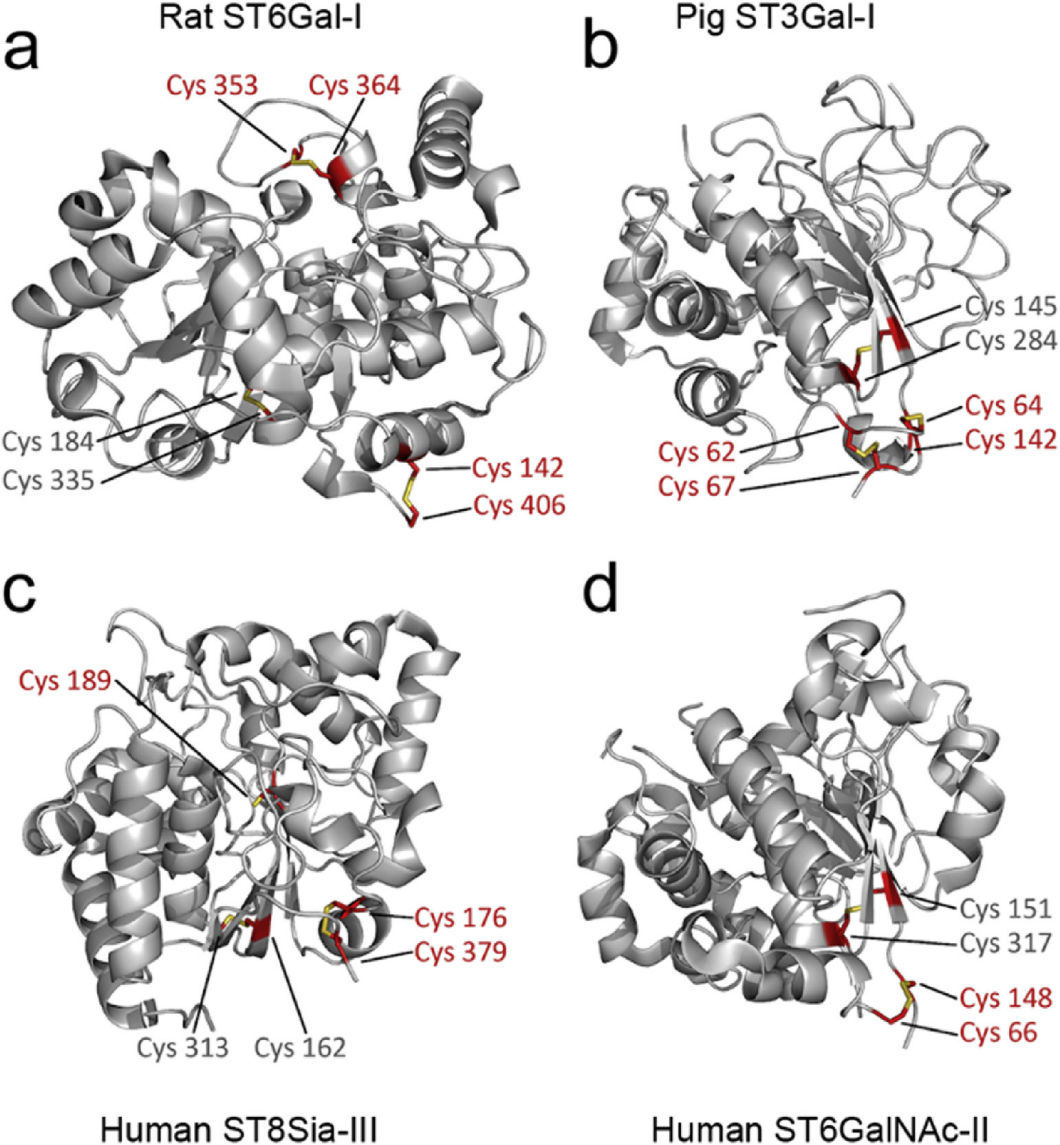
Comparison of the published mammalian sialyltransferase crystal structures and conservation of the surface exposed disulfide bonds within their catalytic domains. The figure shows conserved cysteine residues forming both structure-associated (grey text) and surface exposed (red text) disulfide bonds. For further details, see the text. The structures shown depict the following PDB database identification codes: **a)** PDB entry 4MPS. **b)** PDB entry 2WNF. **c)** PDB entry 5BO7. **d)** PDB entry 6APL.

Structure comparisons of the known sialyltransferase crystal structures [41,[43], [44], [45]] indicated further that the two surface exposed disulfide bonds are conserved among the members of the sialyltransferase gene family. For example, the structure of the rat ST6Gal-I catalytic domain (Fig. 6a) displays identical disulfide bonds to those present in the human enzyme (Fig. 5a). Likewise, the pig ST3Gal-I (Fig. 5b) sialyltransferase (also known as SIAT4A), which is implicated in mucin type O-glycan, glycosaminoglycan and glycolipid biosynthesis, shows similarly two such surface exposed disulfide bonds, albeit in a different configuration, suggesting their conservation in other sialyltransferases as well. Their presence and likely sensitivity hypoxia is supported by the reduced sialylation of O-glycans in hypoxic cells (Fig. 1a). In contrast, the human ST8Sia-III α2,8-sialyltransferase (Fig. 6c) and human ST6GalNAc-II (Fig. 6d) display only one such surface exposed disulfide bond and may behave differently from the former two. Whether the ST3Gal-III, a sialyltransferase that terminates N-glycans with α-2,3-linked sialic acid behaves similarly to the ST6Gal-I remains unclear until its crystal structure becomes available. Without detailed structural information, it is difficult reliably to predict which cysteine residues in the primary structure will be disulfide-bonded with each other. This is due to the high sequence variability even between functionally similar enzymes.

### 3.5 Inactivation of ST6Gal-I enhances cell proliferation and migration rates

**Fig. 7:**
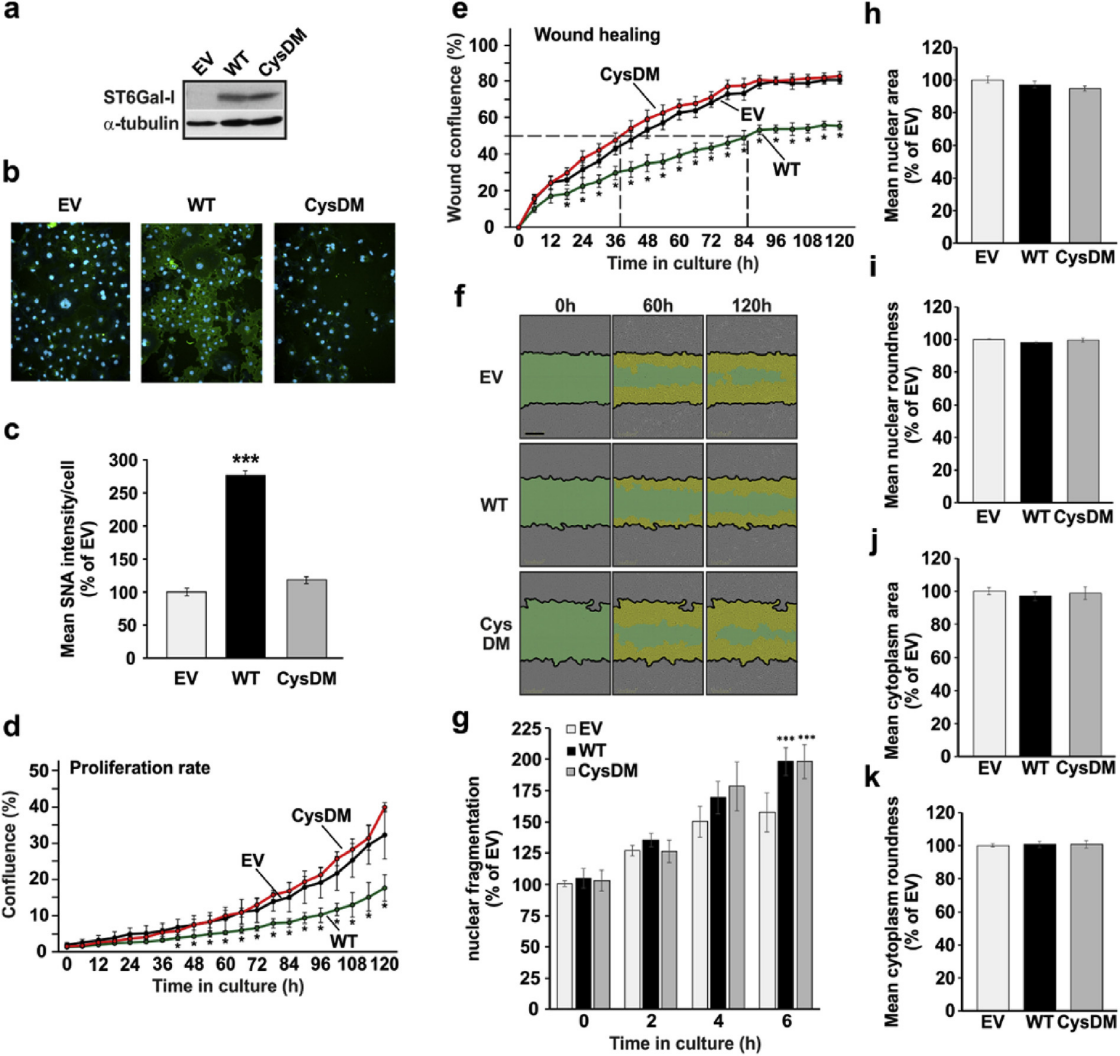
Functional consequences of the inactivation of the ST6Gal-I. a) Immunoblotting of the ST6Gal and its ST6Gal-I_CysDM_ variant in COS-7 cells. Cell transfections were performed by electroporation before SDS-PAGE and immunoblotting with ST6Gal-specific antibodies (anti-CD75) as above. b) Representative figures on SNA lectin binding to the cells transfected separately with the same plasmids. Transfections and lectin staining was carried out as describe above. c) Quantification of the SNA lectin binding to cells using the high content imaging system. The data are shown as relative changes (mean % + SD, n = 3, 70–90000 cells/assay). **d)** Live cell proliferation rate analysis of COS-7 cells transfected with an empty vector (EV) or with vectors expressing the wild-type ST6Gal-I (WT) or the cysteine double mutant of ST6Gal-I (CysDM). Data are expressed as mean confluence % (±SD (n = 5) using a build-in Incyte™ protocol. The star (*) mark the statistically significant difference (p < 0.05) between relative confluence values of the wild-type ST6Gal-I (WT) and the cysteine double mutant (ST6Gal-I_CysDM_) at each time point. **e)** Live cell wound closure analysis of wild type (WT) and double cysteine (CysDM) ST6Gal-I variant expressing cells. The graph shows thee mean confluence (% ±SD (n = 4) at the indicated times after making the wound. Significant changes between confluence values between the wild-type ST6Gal-I (WT) and cysteine double mutant of ST6Gal-I (CysDM) at each time point are marked by stars (p < 0.05*). **f)** Representative images of the wound closure in COS-7 cells transfected with an empty vector (EV), ST6Gal-I (WT) or the cysteine double mutant of ST6Gal-I (CysDM) at 0 h, 60 h and 120 h time point after introduction of the wound. Scale bar 300 μm. Green color represents the original wound, and yellow color depicts the area of repopulated cells. g) Sensitivity cells to staurosporine-induced apoptosis. Cells transfected with the indicated ST6Gal-I variants were subjected to 5 μM staurosporine for 0–6 h. After fixing, cells were stained with the Hoechst dye before imaging high content imaging system (Operetta, PerkinElmer). Fragmentation index of nuclei was then calculated after image segmentation using the “RMS Nuclear Fragmentation” built-in protocol, in which the coefficient of variance percentage (CV%) of the nuclear intensity is used to calculate the fragmentation index. Healthy nuclei have low CV values whereas fragmented nuclei have higher CV values. The values represent the mean (±SD, n = 3, 15,000 cells each. Statistically significant changes are marked with stars (p < 0.05*, p < 0.01**, p < 0.001***). **h-k)** The effect of the wild type ST6Gal-I and its double cysteine variant on nuclear and cell morphology. Cells transfected with the indicated constructs were plated on a 96 well plate, fixed immuno-stained with an α-tubulin antibody, and counterstained with the Hoechst dye. After imaging (Operetta, PerkinElmer) and segmentation, in-built “Harmony” protocols were used to calculate nuclear and cytoplasmic areas (h and j) and their roundness (i and k). The data are expressed as the mean % (±SD, n = 3, 15,000 cells each) of the control values (EV = empty vector). No statistically significant changes were detected between the different ST6Gal-I transfectants (p > 0.05).

Previous studies have indicated a correlation between ST6Gal-I activity and cancer metastasis, while in others such correlation has not been found [46]. There are also other studies, showing that ST6Gal-I overexpression changes cellular phenotype or behavior, e.g. by increasing proliferation, migration, radiation resistance, invasion and metastasis [[47], [48], [49], [50]]. Therefore, it was important to address what functional consequences, if any, inactivation of ST6Gal-I has in the cells. To address this, we transfected cells with either an empty vector, wild type ST6Gal-I (ST6Gal-I_WT_) or the CysDM encoding plasmids, and followed possible changes in proliferation, migration and apoptotic resistance as well as changes in nuclear or cell morphology. We found that overexpression of the ST6Gal-I_WT_, but not that of CysDM (Fig. 7a), increased α-2,6-linked sialic acid level in the cells (Fig. 7b and c), as expected. This increase had a negative impact on cell proliferation (Fig. 7d) and migration in a wound healing assay, while the CysDM had no effect (Fig. 7e and f). None of them, however, altered markedly cells' apoptosis resistance (Fig. 7g), the size of the nuclear area and its roundness (Fig. 7h and i), or cell morphological properties (cytoplasmic area and roundness, Fig. 7j and k). Thus, in contrast to cancers, increased sialylation by overexpressed ST6Gal-I in non-malignant COS-7 cells is inhibitory to cell proliferation and migratory behavior. Therefore, it is likely that inactivation of ST6Gal-I by hypoxia serves for better cell survival by enhancing their proliferative and migratory behavior of hypoxic cells.

## 4 Discussion

The data described above provides a direct mechanistic link between hypoxia, attenuated sialylation of N- and O-glycans, lowered Golgi (and ER) oxidative potential, reduced disulfide bond formation and the loss of catalytic activity of a relevant ST6Gal-I N-sialyltransferase and its ability to interact with its binding partner B4GalT-I. Thereby, hypoxia-induced silencing of the ST6Gal-I provides also a rational explanation for the attenuated sialylation of N-glycans in hypoxic cells. Intriguingly, its binding partner B4GalT-I appears not to be affected, consistent with increased levels of terminal galactoses in N-glycans. Similar surface exposed disulfide bonds are also present in ST3Gal-I sialyltransferase, an enzyme implicated in the synthesis of O-glycans, glycosaminoglycans and glycolipids. This suggests that other glycosylation pathways are also defective in hypoxic cells. Our findings also show that glycosylation can be, to a large extent, independent of the HIF system, and emphasizes the sensitivity of the glycosylation process to changing oxygen concentrations. Since the HIF system involves HIF-dependent transcription, followed by translation of a certain protein, i.e. represents a long-term response towards hypoxia, the involvement of a redox switch regulating directly glycosylation enzymes themselves indicates a short-term response. Hence, this system allows an immediate response towards hypoxia that helps cells to proliferate due to their altered glycosylation profiles.

The data unveils a hitherto unknown regulatory circuit that relies on disulfide bond formation in the Golgi and is needed normally for the catalytic activation of ST6Gal-I in this cellular compartment. In support of this view, we showed that the loss of the two surface exposed disulfide bonds seen in hypoxic cells or obtained by mutagenesis of the selected cysteine residues in ST6Gal-I resulted in the loss of enzymatic activity and its ability to interact with GalT-I. The two surface-exposed disulfide bonds in ST6Gal-I are thus needed not only for the catalytic activity but also to facilitate ST6Gal-I/B4GalT-I complex assembly, a phenomenon that likely involves a subtle conformational change in ST6Gal-I and is mediated by the formation of the two disulfide bonds. The fact that the assembly of the complex takes place only after the enzymes arrive in the Golgi compartment [23], indicates that the two surface-exposed disulfide bonds must also form in this compartment. In accordance with this view, the oxidative potential was reduced by hypoxia to the same level it is in the ER in normoxic cells. The higher oxidative potential of the Golgi lumen, therefore, would provide proper conditions for disulfide bond formation in the Golgi and likely serves as a means to prevent premature sialylation of various acceptor glycans by keeping the enzyme inactive until it reaches the slightly acidic and more oxidative environment in the Golgi lumen. Finally, similar redox state-sensitive disulfide bonds are known to exist in other proteins including occludin, whose homotypic interactions and motility in the membrane are similarly affected by hypoxia [51]. The formation of the von Willebrand factor (vWF) multimers from vWF oligomers takes place in the Golgi compartments and involves tail-to-tail interactions via disulfide bonds [52]. This molecular circuitry involving hypoxia-sensitive disulfide bond formation in the Golgi apparatus, therefore represents a new level of regulation of glycosylation and shows that enzymes can be activated post-translationally in a compartment they normally function in the cell.

Sialylation regulates numerous cellular processes, including cell–cell communication, immune surveillance, defense against pathogens and cancer progression and metastasis [46,53]. In cancers, especially α-2,6-sialylation is increased and correlates with increased cell proliferation, migration, invasion and metastasis, while inhibiting apoptosis [[47], [48], [49], [50]]. In contrast, we found that overexpression of the wild type ST6Gal-I, but not that of the inactive cysteine mutant, slowed down cell proliferation and migration, while having no effect on staurosporine-induced apoptosis and cell or nuclear morphology. Therefore, the inactive ST6Gal-I in hypoxic cells likely helps cells to survive and adapt to low oxygen levels. This discrepant behavior between cancer cells and cells expressing inactive ST6Gal-I likely reflects the lack of truncated O-linked glycans in our experimental system, as cancer cells and also hypoxic cells (this study) display tumorigenesis promoting truncated O-linked glycans [21,54]. Nevertheless, the fact that increased α-2,6-sialylation slowed down cell proliferation and migration suggests that inactivation of ST6Gal-I has an important role in allowing non-malignant cells to adapt to low oxygen environment. It is also of notice that the ST6Gal-I almost certainly is not the only enzyme that is sensitive to hypoxia, as our structure comparisons revealed similar surface exposed disulfide bonds in ST3Gal-I, an enzyme implicated in the synthesis of O-linked glycans, keratan sulfate and glycolipids.

Our findings, however, do not explain why hypoxia alters Golgi redox homeostasis. The main obstacle preventing this is that only few proteins with clear regulatory role in setting up the high oxidative potential in the Golgi lumen are known. One of them is the Golgi-localized QSOX1a protein that has been shown to play a role in disulfide bond formation, possibly in the maturation of ECM components and the formation of higher order structures there [55]. Two other proteins, glutaredoxins Grx6 and Grx7, which are closely related with each other and likely are part of the redox state regulatory system in the Golgi, belong to the glutaredoxin family of proteins that normally catalyze the reduction of disulfide bonds in their substrate proteins. They both localize in the cis-Golgi in *S. cerevisiae* [56] and show high glutaredoxin activity. Yeast knockout cells also display growth defects and sensitivity toward oxidizing agents, indicating their important roles in the maintenance of Golgi redox homeostasis. However, Grx6 and Grx7 do not seem to have a general role in the oxidative protein folding, but rather, in counteracting the oxidation of specific thiol groups in substrate proteins [56]. These findings thus imply that the redox pair discovered in the Golgi may represent another compartment-specific redox pair. Similar redox switches are well known to exist in the cytosol, plasma membrane, nucleus and the extra-cellular space, where the thioredoxin1/Txnip (thioredoxin binding protein-2 (TBP-2)/vitamin D3 upregulated protein (VDUP1)) plays an important role. Another similar system may account for redox homeostasis in mitochondria, where the Trx2/Txnip complex is enriched under conditions of oxidative stress [57]. Further work, therefore, is needed to identify the molecular machinery responsible for the unique redox state and high oxidative potential of the Golgi compartments.

## Author contributions

A.H., F.K-A., E.K., E.Y.D., D.M., D. H. and M.N performed research and analyzed the data, A.H-L. designed the sialyltransferase enzymatic assays, supervised the work of M.N and compiled the activity data. T.G. supervised the structural studies of. D.B. SK together with TK designed the experiments, compiled the data and wrote the manuscript. F.K-A., E.K., A.H-L, and T.G. read the manuscript and made suggestions to the text.

## Conflicts of interest

The authors declare no conflict of interest regarding this publication.
