# Supplementary material for: A Golgi-associated redox switch regulates catalytic activation and cooperative functioning of ST6Gal-I with B4GalT-I
Source: Redox Biol. 2019 Apr 4;24:101182. doi: 10.1016/j.redox.2019.101182 (PMC6454061; doi:10.1016/j.redox.2019.101182)
Supplement: Multimedia component 1 [file mmc1.docx]

**Table EV1.** qPCR primer sequences

| **Gene name** | **EC Number** | **Enzyme** | **Forward (5’-3’)** | **Reverse (5’-3’)** |
| --- | --- | --- | --- | --- |
| *ST6GAL1* | EC:2.4.99.1 | ST6Gal-I | ACCCCAATCAGCCCTTTTACA | CTGGTCACACAGCGTCATCA |
| *B4GALT1* | EC:2.4.1.22 | B4GalT-I | CCAGGCGGGAGACACTATATT | CACCTGTACGCATTATGGTCAT |
| *MGAT1* | EC:2.4.1.101 | GlcNAcT-I | CGCAAGTTCCAGGGCTACTAC | CTTCAGCAGCGGATAGGTGG |
| *MGAT2* | EC:2.4.1.143 | GlcNAcT-II | TGACAACGTCCTCGTCATCTT | CCTGGAAACTCGTTAGGGTACAA |
| *ST3GAL3* | EC:2.4.99.6 | ST3Gal-III | GCCTGCTGAATTAGCCACCAA | GCCCACTTGCGAAAGGAGT |
| *GALNT6* | EC:2.4.1.41 | ppGalNAcT-6 | CTGTTCTCCATAAACCAGTCCTG | CGCTGGCAAAGGCATTGAAA |
| *GCNT1* | EC:2.4.1.102 | C2GNT-1 | AGCGGTATGAGGTCGTTAATGG | GACCACGAAGTAGGCACTGC |
| *GCNT3* | EC:2.4.1.102 | C2GNT-3 | TCTGGGCTGCTATATGCTGC | GTTGATAGACCTCTTTGCTGGAA |
| *GCNT4* | EC:2.4.1.102 | C2GNT-4 | GTTGTGGCAATGACCAGTGAT | AGCATGGATAAGCCTTTCAACC |
| *B3GNT6* | EC:2.4.1.147 | C3GNT-1 | GTGCGCCGCCTCTTTCTATT | CCAGCCAGTCGAGCAAGTG |
| *SIAT4A* | EC:2.4.99.4 | ST3Gal-I | GGAGGACGACACCTACCGAT | CCACCGACCTCTTCTCCAG |
| *ST6GALNAC1* | EC:2.4.99.3 | ST6GalNAc-I | CACAGCCAAGACGCTCATTC | CCTTTCTGTCTCGTCCTTGTTG |
| *C1GALT1* | EC:2.4.1.122 | C1GalT-1 | TCCTCTGTGGATCAGCAATAGG | TTAGGCTGGGTGTCAACCTTT |
| **Control PCR primers: Gene name** | | | **Forward (5’-3’)** | **Reverse (5’-3’)** |
| *LDHA Lactate dehydrogenase A* | | | TTGACCTACGTGGCTTGGAAG | GGTAACGGAATCGGGCTGAAT |
| Enzyme names as listed in the KEGG database (see Http://ww.genome.jp/kegg/pathway.html | | | | |
