# Supplementary material for: A Golgi-associated redox switch regulates catalytic activation and cooperative functioning of ST6Gal-I with B4GalT-I
Source: Redox Biol. 2019 Apr 4;24:101182. doi: 10.1016/j.redox.2019.101182 (PMC6454061; doi:10.1016/j.redox.2019.101182)

Fig. EV1. Hypoxia-induced N- and O-glycosylation changes in COS-7 cells using a lectin microarray analyses. a) The graph shows the raw data values from two different sample sets (normoxia and hypoxia) used for the calculation to get the subtracted glycan fingerprints shown above together with lectin specificities for different sugars as defined in the manufacturers’ web pages.

**
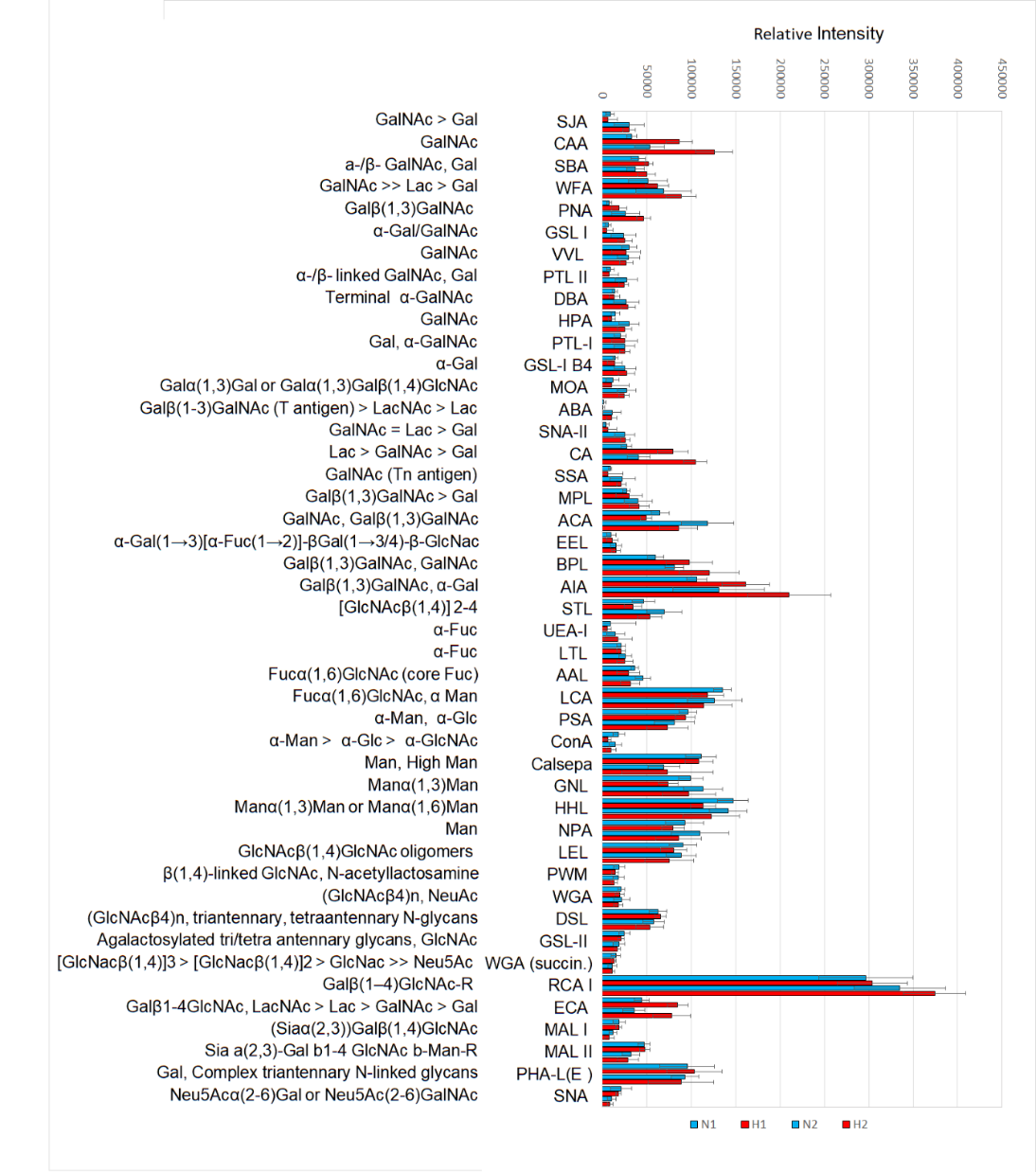
**

**Fig. EV2**. The effect of hypoxia on the assembly of the homomeric glycosyltransferase complexes implicated in the synthesis of N- and O-linked glycans. a) N-glycosyltransferase homomers. b) O-glycosyltransferase homomers. In both cases, cells were transfected with the indicated mVenus- and mCherry-tagged enzyme constructs, cultivated either in normoxic or hypoxic conditions for 20 h before quantification of the FRET signals with the high content imaging system. The calculated FRET efficiencies (%) are presented as mean % (+SD, n=3, 15,000 cells each) of the normoxic control values (set to 100%). Statistically significant changes are marked in the figures with stars (p < 0.05*, p < 0.01**, p < 0.001***).

**
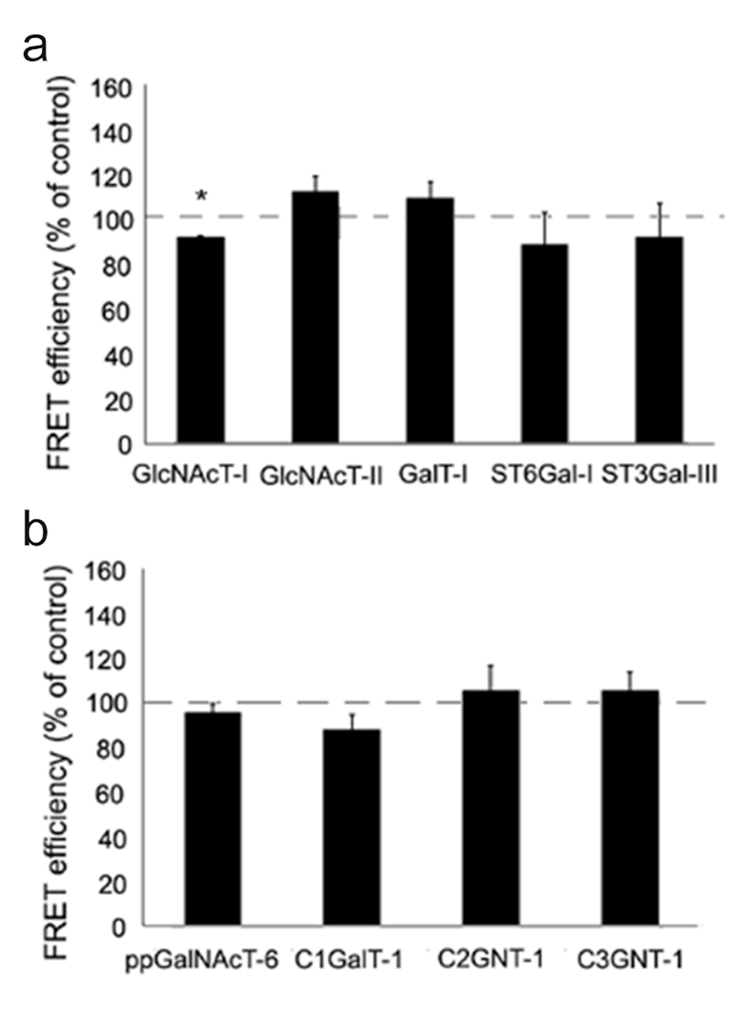
**

**Fig. EV3**. Co-localization of ST6Gal-I with the Golgi marker GM130 in DTT treated cells. Cells were treated with DTT for 20 h post-transfection before fixation and staining the anti-GM130 antibody and Alexa594-conjugated anti-mouse secondary antibody. Note that DTT does not cause mis-localization of the ST6Gal-I even after 20 h treatment.

**
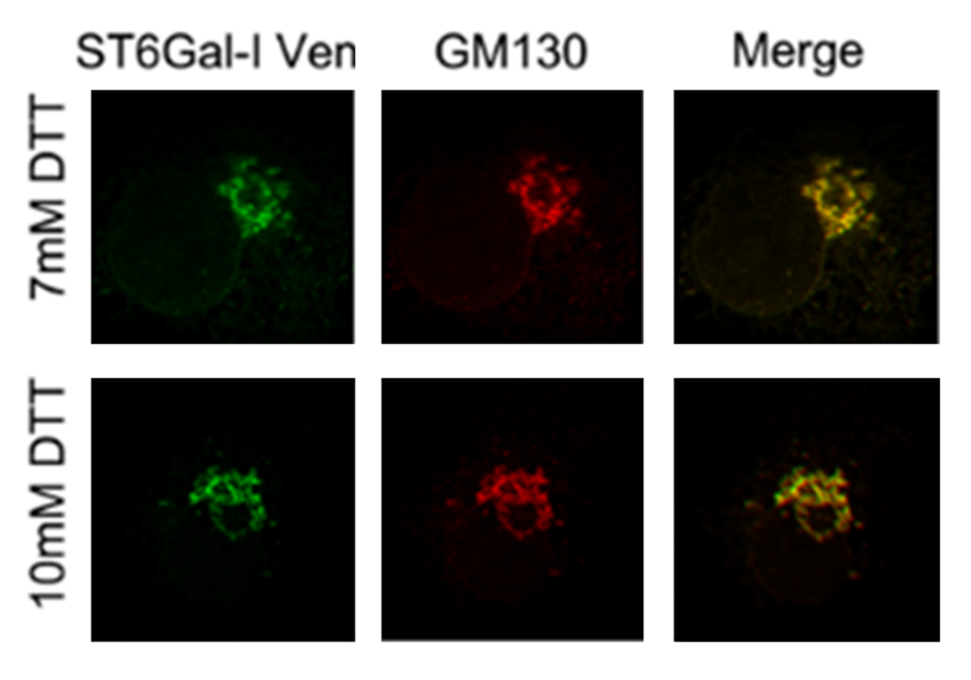
**

**Fig. EV4.** Quantification of the ST6Gal-I protein levels in cells transfected with the depicted ST6Gal-I constructs. In brief, 48 h post-transfection, the cell lysates were subjected to SDE-PAGE and immunoblotting with the anti-ST6Gal-I antibody (anti-CD75, Acris). Band intensities in the blots were quantified using the ImageJ software and used first to normalize the ST6Gal-I protein levels using α-tubulin as a loading control. Normalized enzyme levels (WT/CysDM ratio) were then used to normalize SNA binding intensities/cell (Fig. 4f). Similar blots from cells transfected identically were also used for normalization of ST6Gal-I enzyme activities (Fig. 4g).


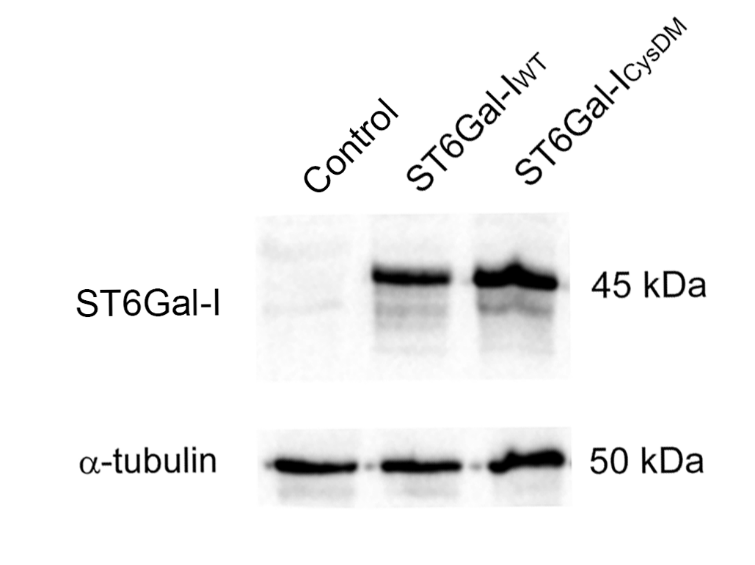

Supplement: Multimedia component 2 [file mmc2.docx]
